# Effects of attention manipulations on motivated attention to feared and nonfeared negative distracters in spider fear

**DOI:** 10.1186/1471-2202-14-139

**Published:** 2013-11-09

**Authors:** Joakim Norberg, Stefan Wiens

**Affiliations:** 1 Department of Psychology, Stockholm University, Stockholm S-106 91, Sweden; 2 Stockholm Brain Institute, Karolinska Institutet, Retzius väg 8, Stockholm S-171 76, Sweden

## Abstract

**Background:**

When people view emotional and neutral pictures, the emotional pictures capture more attention than do neutral pictures. In support, studies with event-related potentials have shown that the early posterior negativity (EPN) and the late positive potential (LPP) to emotional versus neutral pictures are enhanced when pictures are attended. However, this motivated attention decreases when voluntary attention is directed away from the pictures. Most previous studies included only generally emotional pictures of either negative or positive valence. Because people with spider fear report intense fear of spiders, we examined whether directing attention away from emotional pictures at fixation decreases motivated attention less strongly for spiders than for generally negative distracters.

**Results:**

We recorded event-related potentials from 128 channels to study whether manipulations of attention (i.e., spatial attention and perceptual load) decrease the EPN and the LPP to emotional distracters less strongly for spiders than for fear-irrelevant negative pictures in people with spider fear. Results confirmed that the EPN and the LPP to spiders (vs. neutral pictures) were particularly enhanced in participants with spider fear compared to participants without spider fear. When attention was directed away from the pictures, the EPN and the LPP to spiders (vs. neutral pictures) decreased similarly in fearful and nonfearful participants. Further, in fearful participants, the decrease in the EPN and the LPP was similar for spiders and for fear-irrelevant negative pictures.

**Conclusions:**

Our findings suggest that for people with spider fear, directing attention away from emotional pictures at fixation decreases motivated attention to these distracters similarly for spiders as for fear-irrelevant negative pictures. These findings imply that attention to spiders in spider fear does not exceed the level of attention expected from the spider pictures’ high arousal and negative valence (i.e., their intrinsic motivated attention).

## Background

Because emotional stimuli are motivationally relevant, they capture attention. This idea is common to theories such as *motivated attention*[1], *emotional attention*[2], and *natural selective attention*[3]. According to the motivational model of emotion [1,4], emotional stimuli such as predators, food, or sexual scenes activate either the defensive or appetitive motivational system, thus preparing the organism for appropriate action. Which system (defensive or appetitive) is activated corresponds to the subjective experience of valence, and the degree of activation corresponds to the subjective experience of arousal [1,4].

Allocation of attention to emotional pictures has been studied by means of event-related potentials (ERPs). Two commonly used measures are the early posterior negativity (EPN) and the late positive potential (LPP). Both reflect the stronger allocation of attentional resources to emotional than neutral pictures [5,6]. When the ERP waves to neutral pictures are subtracted from the ERP waves to emotional pictures (e.g., mutilation or erotica), the early posterior negativity (EPN) is a negative deflection starting about 200 ms after picture onset and is evident over electrodes in the temporal-occipital region [7]. Similarly, the ERP waves to the pictures reveal a late positive potential (LPP) that is seen over parietal-central electrodes from about 300 ms after stimulus onset and that is larger for emotional than neutral pictures [8]. Both the EPN and the LPP can be observed without explicit instruction to attend to the emotional content [5]. Studies on the neural mechanisms have found correlations between LPP amplitudes to emotional pictures (of negative or positive valence) and fMRI activations in the lateral occipital, inferotemporal, and parietal visual areas [9] and in the insula, anterior cingulate, ventral striatum/nucleus accumbens, and amygdala [10]. Also, a recent combined EEG/fMRI study suggests that these different areas contribute differently to LPP amplitudes depending on valence [11]. In sum, these findings for the EPN and LPP to emotional (vs. neutral) pictures show that emotional stimuli capture attention without explicit instruction and thus, are intrinsically motivational.

The stronger allocation of attentional resources to emotional than neutral pictures, as indexed by the EPN and LPP, has also been examined in phobia. Phobia is the most common anxiety disorder, with a lifetime prevalence ranging from 10% to 18% [12,13]. The most common phobia is fear of spiders, with a prevalence of 1.2% for men and 5.6% for women [14]. Phobia is usually thought to be the result of aversive conditioning [15], that is, Pavlovian or classical conditioning where a biologically innate fear response is coupled with a stimulus that does not by itself elicit it. In support, brain imaging show similar activation patterns for phobia and experimentally induced aversive conditioning in humans [16].

ERP studies have confirmed that feared pictures capture attention. In people who are diagnosed with spider phobia or who report high levels of spider fear, the LPP amplitudes are enhanced to spiders versus neutral pictures [17-26], as are EPN amplitudes [23,27].

These ERP findings of larger EPN and LPP amplitudes to spiders than to neutral pictures in participants with high levels of spider fear (or phobia) are relevant to theories that argue that attentional biases to threat are important factors in the development of anxiety disorders [28,29]. However, it is unclear whether the ERP findings mean that in people with spider fear, attention to spider pictures exceeds the level of attention expected from the spider pictures’ high arousal and negative valence (i.e., their intrinsic motivational value). To study the effect of spider pictures on attention over and above the level of attention that is expected from the pictures’ high arousal and negative valence, it is necessary to compare spider pictures to fear-irrelevant, negative pictures in people with spider fear [28]. Specifically, if people with spider fear rate spiders and other negative pictures similarly in terms of valence and arousal but attend more strongly to the spiders than to the other negative pictures, then this finding would demonstrate that attention to spider pictures in people with spider fear exceeds the level of attention expected from the spider pictures’ high arousal and negative valence. Unfortunately, for the LPP, studies did not analyze arousal and valence ratings across picture types [23,26], or studies did not match picture types in these regards [19,22]. For the EPN, studies did not report whether emotion ratings differed between the picture types [23]. Also, another study on the EPN compared spider pictures that had mainly simple picture composition (i.e., figures) with other negative pictures that had mainly complex composition (i.e., scenes) [27]. However, this design confounds emotion with picture composition because the EPN is smaller to emotional scenes than to emotional figures even if valence and arousal are matched [30]. Taken together, although there is strong evidence that emotional pictures draw attention, as indexed by the EPN and the LPP, it is unresolved whether the effect on attention from spider pictures in people with spider fear exceeds the level that would be expected on the basis of the spider pictures’ intrinsic motivational relevance (i.e., high arousal and negative valence).

To study whether emotional pictures capture attention even if the pictures are task irrelevant (i.e., distracters), many studies have investigated emotional responses during spatial inattention, that is, by comparing responses to pictures in attended with those in unattended locations [31,32]. The relevant ERP studies on this issue have mainly used emotional pictures from the International Affective Picture System (IAPS) [33]. Results showed that LPP amplitudes to negative and positive IAPS pictures and pictures of emotional faces were eliminated when the pictures were presented in the periphery and attention was directed at fixation [34-37], and strongly reduced if not eliminated when pictures were presented at fixation and attention was directed to the periphery [30,38-43]. Similar results were reported for studies on the EPN with reductions of amplitudes when emotional pictures were presented in unattended locations [30,34], even though some studies reported null findings [40-42]. However, it has yet to be studied whether spatial inattention decreases the EPN and the LPP to spiders in spider fear. Also, it is unresolved whether effects of spatial inattention decrease the EPN and the LPP less strongly for spiders than for fear-irrelevant negative pictures in spider fear. If so, this finding would suggest that attention is drawn to the feared spiders per se over and above their intrinsic motivational value (i.e., high arousal, negative valence).

Effects of distracting emotional pictures on attention can also be studied by presenting task-irrelevant stimuli while participants perform a concurrent task that varies in attentional demands. According to Load theory [44,45], attention is a resource that can be distributed across tasks. If a task is taxing only a small amount of this resource (low perceptual load), there are spare resources that are used to process task-irrelevant stimuli. However, if a task is consuming all resources (high perceptual load), there are no resources left and consequently no task-irrelevant stimuli will be processed. Notably, a recent study with spider fear suggested that Load theory may not apply fully to spider pictures in spider fear [21]. In this study, participants with and without high levels of spider fear were shown task-irrelevant pictures of spiders and mushrooms at fixation while participants performed a discrimination task on letters that surrounded the pictures with either three letters (low load) or six letters (high load). Results showed that enhanced LPP amplitudes to spider pictures in fearful participants did not differ between low and high load (with negligible effect size). Thus, perceptual load did not seem to influence processing of spiders in spider fear.

However, several subsequent studies reported similar null effects for generally negative pictures from the IAPS picture system (e.g., mutilation, disgust) [40,41]. Participants performed a letter discrimination task while pictures were shown at fixation. Performance on the letter discrimination task decreased strongly with load. Although participants showed generally enhanced EPN and LPP amplitudes to negative pictures, the EPN and LPP amplitudes did not differ between low and high load. Similarly, when participants performed either a detection task (low load) or a discrimination task (high load) on symbols at fixation, the steady-state visual evoked potentials (ssVEP) to flickering negative IAPS pictures in the background were unaffected by load [46]. In contrast to these null findings, one study found that the EPN to IAPS pictures (positive and negative valence) versus neutral pictures decreased when task demands increased [47]. In the study, lines were superimposed on the pictures, and participants had to count the number of line trials. Task demands were increased by increasing the proportion of line trials. If it is argued that this manipulation increases perceptual load, these findings suggest that perceptual load reduces EPN to generally emotional pictures. However, spiders and other negative pictures in participants with high spider fear have not been included in the same study. Therefore, it is possible that in spider fear, effects of perceptual load decrease the EPN and the LPP less strongly for spiders than for fear-irrelevant negative pictures in spider fear, thus suggesting that attention is drawn to the feared spiders per se.

To summarize, theories on the etiology of anxiety disorders assign an important causal role to attentional biases [28,29]. Previous studies confirm that people with high spider fear attend more strongly to spiders than to neutral pictures, as indexed by enhanced EPN and LPP amplitudes to spiders versus neutral pictures. Further, previous research has studied effects of manipulations of spatial attention and perceptual load on EPN and LPP amplitudes for generally negative pictures. However, no previous study has studied whether effects of manipulations of spatial attention and perceptual load differ for spiders and other negative pictures in people with high spider fear. If the EPN and LPP amplitudes are reduced less strongly for spiders than for other negative pictures in people with high levels of spider fear, then this finding would provide evidence that in spider fear, spiders have an effect on attention over and above their intrinsic motivational value (i.e., high arousal and negative valence). To address this question, the present study included participants with and without spider fear and recorded ERPs to spiders, negative pictures, and neutral pictures during manipulations of spatial attention and perceptual load. Attention to the pictures was indexed by the EPN and LPP. Because the EPN and LPP are affected by picture composition [30,48,49], the different picture categories (spiders, negative pictures, and neutral pictures) were matched in picture composition to avoid any confounding effects from picture composition.

## Methods

### Participants

Participants were recruited by word of mouth or e-mail to students at the Department of Psychology at the Stockholm University. The study was approved by the regional ethics board in Stockholm, Sweden (2008/1464-31/5). Before the experiment, participants gave written informed consent. After the experiment, they received course credits or movie vouchers. Participants were screened for spider fear with a Swedish version of the German Spider Anxiety Screening [50]. This questionnaire consists of four 6-point items that sum up to a maximum of 24 points. Those who scored more than 17 points (fearful, *n* = 17) or less than 3 points (controls, *n* = 17) were recruited for the study. At the time of the experiment, participants also filled out questionnaires on spider fear (SPQ) [51], snake fear (modeled after [50]), disgust sensitivity [52], trait anxiety (STAI trait anxiety inventory) [53], and affect (positive and negative affect of PANAS) [54] before and after the experiment.

Participants were interviewed before the experiment by a clinical psychologist with the phobia part of the ADIS structured interview [55]. Three participants who showed high spider fear during the initial screening were excluded from the final sample because they did not qualify as spider fearful in the interview and when filling in the SPQ before the actual experiment (SPQ < 7). In the remaining sample, participants in the spider fear group fulfilled criteria for phobia according to DSM-IV [56], except that most of them claimed not being bothered by their excessive fear of spiders.

Three participants were excluded because of equipment failure or excessive noise in the EEG recordings. In the final sample of 13 participants with spider fear and 15 participants without spider fear, the percentage of females were larger in the fear group than in the no-fear group (61% vs. 39%), χ2(1, *n* = 28) = 4.37, *p* = .037. However, all analyses were also run checking for interactions with gender, but none were found.

### Stimulus material and procedures

Negative (*n* = 70) and neutral (*n* = 70) color pictures were selected from the 2008 IAPS set [33]. Negative pictures included medical procedures, dead animals, guns and mutilated bodies, whereas neutral pictures included people with neutral facial expression, cars, mushrooms and other objects. Compared to the IAPS normative ratings for neutral pictures, normative ratings for negative pictures were less pleasant (*m* = 2.83 vs. *m* = 5.06), *t*(138) = 21.61, *p* = .001, *η*^2^_   p_ = .77 and more arousing (*m* = 5.80 vs. *m* = 3.56), *t*(138) = 15.42, *p* = .001, *η*^*2*^_   *p*_ = .63. Color pictures of spiders (*n* = 70) were chosen from the internet.

Picture types were matched in composition [48] based on ratings in a pilot experiment (*n* = 9), *F*(2, 207) = 2.48, *p* = .086, *η*^*2*^_   *p*_ = .023. On a scale from 1 (= figure) to 9 (= scene), composition for neutral IAPS pictures was *M* = 2.14, *SD* = .30; negative IAPS pictures, *M* = 2.26, *SD* = .44; spider pictures, *M* = 2.16, *SD* = .28.

Presentation 13.0 (Neurobehavioral Systems, Inc., Albany, CA) was used to show pictures on a 21-inch View Sonic P227f cathode ray-tube monitor at a 100-Hz refresh rate with a resolution of 1280 × 1024 pixels. Pictures had a size of 17.5 × 14.5 cm (visual angle 12.5° × 10.4°) cm and were centered on a black screen background.

To study effects of manipulations of attention (spatial attention and perceptual load), participants performed four tasks (picture, one-letter, three-letter, and six-letter), presented in separate blocks. Task order was counterbalanced across participants. All blocks contained 210 trials and were preceded by 10 practice trials.

Participants were seated in front of a screen with their heads in a chin rest to make sure they were at a distance of 80 cm from the screen. On each trial, a fixation cross was first presented in the center of the screen (randomly for 800, 900, or 1000 ms), followed by the picture stimulus (70 spider, 70 negative IAPS, and 70 neutral IAPS) (200 ms), which was in its turn followed by a blank screen (1300 ms). The four tasks did not differ in instructions except for the expected spatial locations of the targets. For the picture task, the participants were instructed to attend to the pictures and to perform a speeded letter detection task by pressing the space key whenever the letter X or N (uppercase letters shown in gray Arial font and size 46) was presented superimposed on them (20% of trials; either X or N was randomly selected). To ensure that the task would be easy and that eye gaze was not externally drawn to the location of the letters, two Xs or two Ns were always presented simultaneously on target trials, one in the upper right quadrant and one in the lower left quadrant of the picture (or vice versa). Because differences between conditions in physical low-level properties could confound ERP results, the trials during the picture task were made similar to the trials during the letter tasks by also presenting letters (one, three, or six) surrounding the pictures (uppercase shown in gray Arial font and size 46). For the picture task, participants were instructed to ignore these letters (that did not include X or N).

In the three letter tasks (one-, three-, and six-letter), presentation parameters and the speeded letter detection task were similar as those in the picture task with the following exception: Participants were instructed to keep their gaze on the position of the fixation cross but to ignore the pictures and instead focus their attention on the letters surrounding them. Perceptual load was manipulated by changing the number of letters that were presented around the picture: from low load (one-letter task) to medium load (three-letter task) to high load (six-letter task). The letters could take six possible positions around the pictures (two above the picture, two below, one to the right, and one to the left). Target letters were N and X (50% of each across all target trials), and distractor letters were H, K, M, Z, W, or V. Participants were instructed to press the space key whenever an X or N was present among the letters surrounding the picture (20% of trials; X or N and its position was determined randomly). Participants should respond as fast as possible without compromising accuracy. On each trial, the distracter letters and their positions were chosen randomly without replacement.

After these four tasks, participants completed a viewing task in which pictures were viewed and rated individually on valence and arousal while ERPs were recorded. Participants viewed and rated each of the 210 pictures. Each trial began with a fixation cross presented randomly between 1000 and 1200 ms, followed by a picture for 200 ms. After 1300 ms, participants had unlimited time to rate valence and then arousal on a computerized version of the Self-Assessment Manikin [57]. Pictures were shown in three blocks of 70 pictures each. Picture order was random except that no more than two pictures of the same picture type were shown consecutively. Although these ERP results will be presented elsewhere, the valence and arousal ratings are included below because of their relevance for the present results.

### Data recording and analysis

For each task, trials on which participants pressed the space key (to indicate that either X or N was shown) were coded as correct responses (hits) or incorrect responses (false alarms). On the basis of the hit rates and the false alarm rates, performance was indexed by *d*’ , which is a signal detection measure of discrimination ability [58]. Performance was also indexed by the mean reaction time to hits.

EEG activity was recorded with an Active Two Biosemi system (BioSemi, Amsterdam, The Netherlands) from 128 sites according to the ABC system at 512 Hz sampling rate with a 104 Hz built in high cut-off filter and an offline notch filter at 50 Hz. For offline processing, BESA (version 5.3.7, MEGIS Software GmbH, Gräfelfing, Germany, http://www.BESA.de) was used. Data were average referenced, that is, each electrode was referenced to the arithmetic average of all 128 electrodes. Noisy electrodes were interpolated with spherical splines. Eye blinks were corrected with a built-in algorithm (15 surrogate brain sources) [59]. Epochs ranged from −100 ms before picture onset to 700 ms and were baseline corrected for the 100 ms before picture onset.

Because motor activity results in artifacts in the EEG recordings, the trials that required responding (i.e., 20% of trials) were excluded from the ERP analyses. So, of the 70 trials for each picture type (neutral, negative, spiders) in each task, only 56 were considered for ERP analysis. Of these, trials with false alarms (i.e., when subjects responded incorrectly) were also excluded. Further, epochs with excessive artifacts were excluded. That is, for each participant, trials were sorted in regards to the amplitude range (max–min) within an epoch, and in regards to the maximum amplitude difference between adjacent data points within an epoch. Then, trials that showed large responses on many channels were excluded. This artifact procedure was conducted over all trials and thus, blind to the actual experimental condition of each trial. Also, this procedure was adjusted individually for each participant to maximize the number of retained trials while minimizing the potentially distorting effect of extreme epochs [60]. Across participants, the mean number of trials per condition ranged between 51.0 and 54.5 (the possible maximum was 56). An ANOVA of the mean number of trials with group (spider fear, no fear), picture type (neutral, negative, spider), and task (picture, one-letter, three-letter, six-letter) showed a main effect of task, *F*(3, 78) = 3.19, *p* = .046, *η*^*2*^_  *p*_ = .109. This effect indicated that the mean number of trials decreased slightly over the four tasks; the means for the picture, one-letter, three-letter, and six-letter tasks were 54.0, 54.3, 52.6, and 51.4, respectively. The ANOVA also showed an interaction between group and picture type, *F*(2, 52) = 3.18, *p* = .050, *η*^*2*^_   *p*_ = .109. The data suggested that relative to nonfearful participants, fearful participants had fewer valid trials for spiders and neutral pictures but not for negative pictures. Notably, there was no interaction between group and picture type (spiders and neutral), *F*(1, 26) < 1, *p* = .362, *η*^*2*^_   *p*_ = .032. These findings provide no evidence that the results for mean amplitudes (reported below) may be confounded by differences in the number of valid trials. Also, in contrast to peak amplitudes, mean amplitudes (as used in our study) are robust against variations in number of trials [61].

To identify EPN and LPP, separate ERP difference waves were computed between negative and neutral pictures across participants and also between spiders and neutral pictures for fearful participants. Because topographical analyses [62] of the EPN and LPP did not suggest any systematic differences between these two conditions, EPN and LPP were defined similarly for all conditions. The EPN was apparent between 180 and 280 ms at 16 electrodes (A10-15, A26-28, B7-11, D31-32; in 10/20 notation: P7-P8, PO7-PO8, O1-O2, O9-O10, PO9-PO10), and mean amplitudes were computed across this interval and electrodes. The LPP was apparent between 300 and 700 ms at 11 electrodes (A1-5, A19, A32, B1-2, D15-16; in 10/20 notation: Cz, CP1- CP2, P1- P2, CPz, Pz).

The main ANOVA included group (spider fear, no fear), picture type (neutral IAPS, negative IAPS, spider), and task (picture, one-letter, three-letter, six-letter). Note that the task effect has four levels and involves effects of spatial attention (i.e., picture vs. one-letter) and of perceptual load (one-, three-, and six-letter). To maximize power in detecting an effect of manipulations of attention (i.e., spatial attention and perceptual load), an a priori contrast was conducted with only the extreme task conditions (i.e., picture and six-letter) to determine whether the groups differed in their responses to spiders relative to neutral pictures. If the task effect was significant, we conducted follow-up ANOVA contrasts on spiders versus neutral pictures to separate effects of spatial attention (i.e., picture vs. one-letter) and of perceptual load (one-letter, three-letter, six-letter). Further, to test specifically whether in participants with spider fear, task effects had less of an effect on spiders than on other negative pictures, we analyzed only in fearful subjects whether the mean amplitude changes from the picture condition to the six-letter condition differed between spiders and other negative pictures. Because our main interest was group differences, we focus on the results of the ANOVAs and *t* tests that pertain to effects involving group or picture type. Both *F* and *p*-values are reported after Greenhouse-Geisser correction and were considered significant if they were below an alpha level of .05, two-tailed. Observed effect sizes are reported as partial eta squared.

## Results

### Early posterior negativity (EPN)

**Figure 1:**
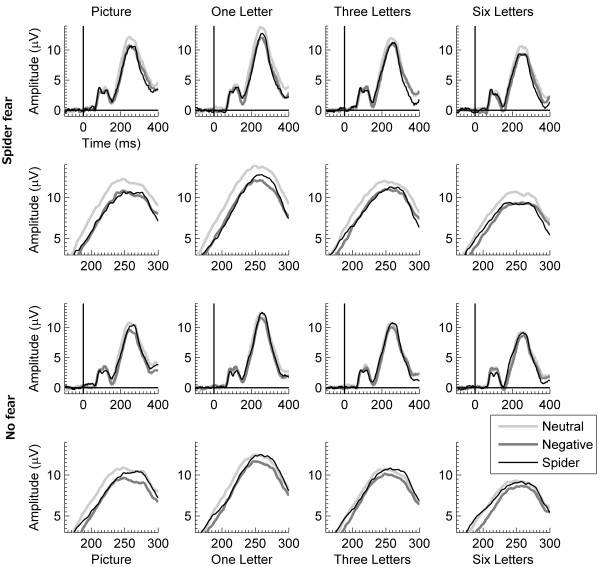
**ERP waves for the EPN.** Mean ERP waves across 16 parietal-occipital electrodes relevant for the early posterior negativity (EPN) for neutral, negative, and spider pictures during the four tasks, separately for participants with spider fear (two top rows) and for participants without spider fear (two bottom rows). For each group, the first row shows the ERP waves between −100 and +400 ms, and the second row shows a blow up of the same ERP waves between +150 and +300 ms.

**Figure 2:**
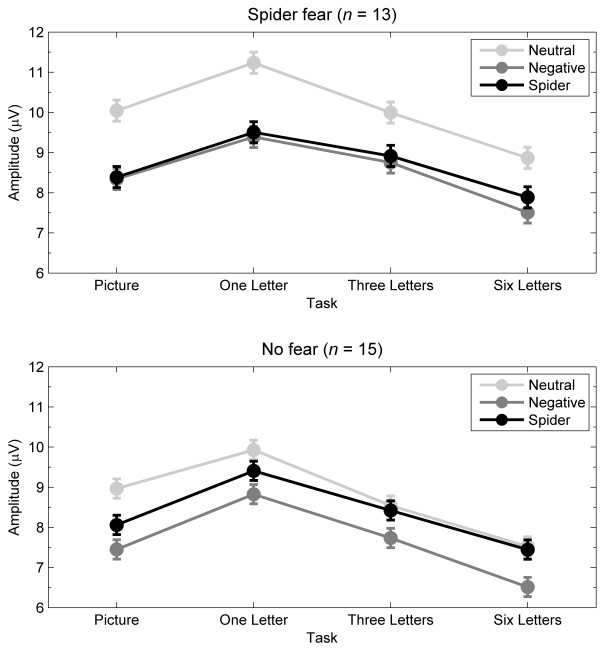
**Mean ERP amplitudes for the EPN.** Mean ERP amplitudes across 180 to 280 ms and across 16 parietal-occipital electrodes relevant for the early posterior negativity (EPN) for neutral, negative, and spider pictures during the four tasks, separately for participants with and without spider fear. Error bars refer to the *SEM* derived from *MSE* within each group.

Figure 1 shows ERP waves for the EPN-relevant electrodes and interval. Figure 2 shows the EPN-relevant mean amplitudes to picture type and task, separately for participants with and without spider fear. Table 1 also shows these mean amplitudes. The ANOVA with group (spider fear, no fear), picture type (neutral, negative, spider), and task (picture, one-letter, three-letter, six-letter) showed mainly an interaction between group and picture type, *F*(2, 52) = 5.04, *p* = .011, *η*^*2*^_   *p*_ = .162. This interaction was caused by less positive amplitudes to spiders (vs. neutral pictures) for fearful than for nonfearful participants, *F*(1, 26) = 9.53, *p* = .005, *η*^*2*^_   *p*_ = .268. This finding indicates that the EPN (i.e., early posterior negativity) for spiders was larger for fearful than nonfearful participants. In contrast, the EPN for negative (vs. neutral) pictures showed no group differences, *F*(1, 26) = 2.50, *p* = .13, *η*^*2*^_   *p*_ = .088, and only a general effect across participants, *F*(1, 26) = 95.72, *p* = .001, *η*^*2*^_   *p*_ = .786.

**Table 1:** Mean ERP amplitudes and picture ratings

	**Spider fear (*n *= 13)**			**No fear (*n *= 15)**		
	**Neutral**	**Negative**	**Spiders**	**Neutral**	**Negative**	**Spiders**
EPN picture task	10.04 (5.97)	8.34 (5.53)	8.39 (5.36)	8.96 (3.52)	7.45 (3.06)	8.06 (2.84)
EPN 1-letter task	11.23 (5.69)	9.39 (4.91)	9.50 (5.39)	9.93 (3.69)	8.82 (3.53)	9.41 (3.15)
EPN 3-letter task	9.99 (5.92)	8.75 (5.83)	8.91 (5.56)	8.54 (3.23)	7.73 (3.11)	8.42 (3.11)
EPN 6-letter task	8.87 (4.98)	7.51 (4.98)	7.88 (5.12)	7.52 (3.02)	6.51 (2.77)	7.44 (3.09)
LPP picture task	−0.40 (1.99)	1.52 (2.31)	1.82 (2.28)	0.69 (1.33)	2.26 (2.50)	1.57 (1.47)
LPP 1-letter task	0.71 (1.45)	1.60 (2.34)	2.04 (1.84)	1.98 (1.92)	3.02 (2.18)	2.44 (1.94)
LPP 3-letter task	0.55 (1.68)	0.93 (1.49)	1.76 (1.52)	1.78 (1.23)	2.02 (1.85)	2.04 (1.65)
LPP 6-letter task	0.53 (1.48)	1.37 (1.69)	1.45 (1.74)	0.94 (1.46)	1.47 (1.67)	1.37 (1.60)
Valence ratings	5.37 (0.43)	3.73 (0.49)	3.28 (0.89)	5.24 (0.27)	3.55 (0.52)	5.05 (0.96)
Arousal ratings	3.89 (1.36)	5.39 (1.13)	6.21 (1.01)	4.01 (1.17)	5.54 (1.22)	4.19 (1.82)

Mean (and *SD*) EPN and LPP amplitudes (in μV) and valence and arousal ratings during the four task conditions for neutral, negative, and spider pictures, separately for participants with and without spider fear. For picture ratings, the scales ranged from 1 to 9: For valence, 1 = unpleasant and 9 = pleasant; and for arousal, 1 = low and 9 = high.

The ANOVA showed no three-way interaction between group, picture type, and task, *F*(6, 156) < 1, *p* = .95, *η*^*2*^_   *p*_ = .007, and also no two-way interaction between picture type and task, *F*(6, 156) = 1.92, *p* = .11, *η*^*2*^_   *p*_ = .069. However, in the specific contrast of task with only the picture condition and the six-letter condition (to maximize sensitivity in detecting an effect of attention), the EPN to spiders (vs. neutral pictures) decreased from the picture condition to the six-letter condition, *F*(1, 26) = 6.52, *p* = .02, *η*^*2*^_   *p*_ = .200, but this effect did not vary with group, *F*(1, 26) < 1, *p* = .80, *η*^*2*^_   *p*_ = .003. Although these findings suggest a combined effect of spatial attention and perceptual load, an ANOVA (of spatial attention) with only the picture condition and the one-letter condition was not significant, *F*(1, 26) < 1, *p* = .69, *η*^*2*^_   *p*_ = .006, and an ANOVA (of perceptual load) with only the one-letter, three-letter, and six-letter conditions was also not significant, *F*(2, 52) = 2.61, *p* = .09, *η*^*2*^_   *p*_ = .091.

Last, we analyzed in fearful subjects whether the mean amplitude changes from the picture condition to the six-letter condition differed for spiders and for negative pictures. In fearful subjects, mean amplitudes between tasks changed similarly for spiders as for negative pictures, *F*(1, 12) < 1, *p* = .38, *η*^*2*^_   *p*_ = .066. That is, the mean EPN to spiders (vs. neutral pictures) was −1.65 during the picture condition and −0.98 during the six-letter condition, and the mean EPN to negative pictures (vs. neutral pictures) was −1.70 during the picture condition and −1.36 during the six-letter condition. Thus, the mean EPN amplitudes decreased from the picture condition to the six-letter condition for spiders (−1.65 minus −0.98 = −0.67) and for negative pictures (−1.70 minus −1.36 = −0.34), and the mean difference in amplitude changes between spiders and negative pictures was −0.33 μV (95% CI = −1.11 to 0.45). Note that the direction of this trend suggested that, if anything, manipulations of attention in fearful subjects *reduced* the EPN more strongly for spiders than for negative pictures.

### Late positive potential (LPP)

**Figure 3:**
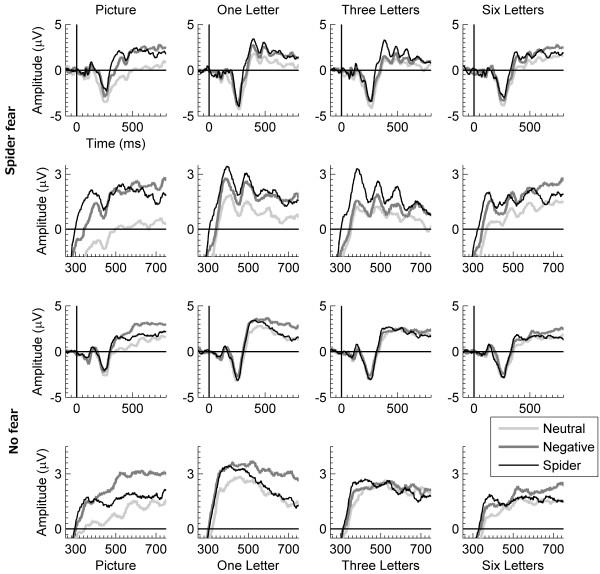
**ERP waves for the LPP.** Mean ERP waves across 11 central-parietal electrodes relevant for the late positive potential (LPP) for neutral, negative, and spider pictures during the four tasks, separately for participants with spider fear (two top rows) and for participants without spider fear (two bottom rows). For each group, the first row shows the ERP waves between −100 and 800 ms, and the second row shows a blow up of the same ERP waves between +250 and +750 ms.

**Figure 4:**
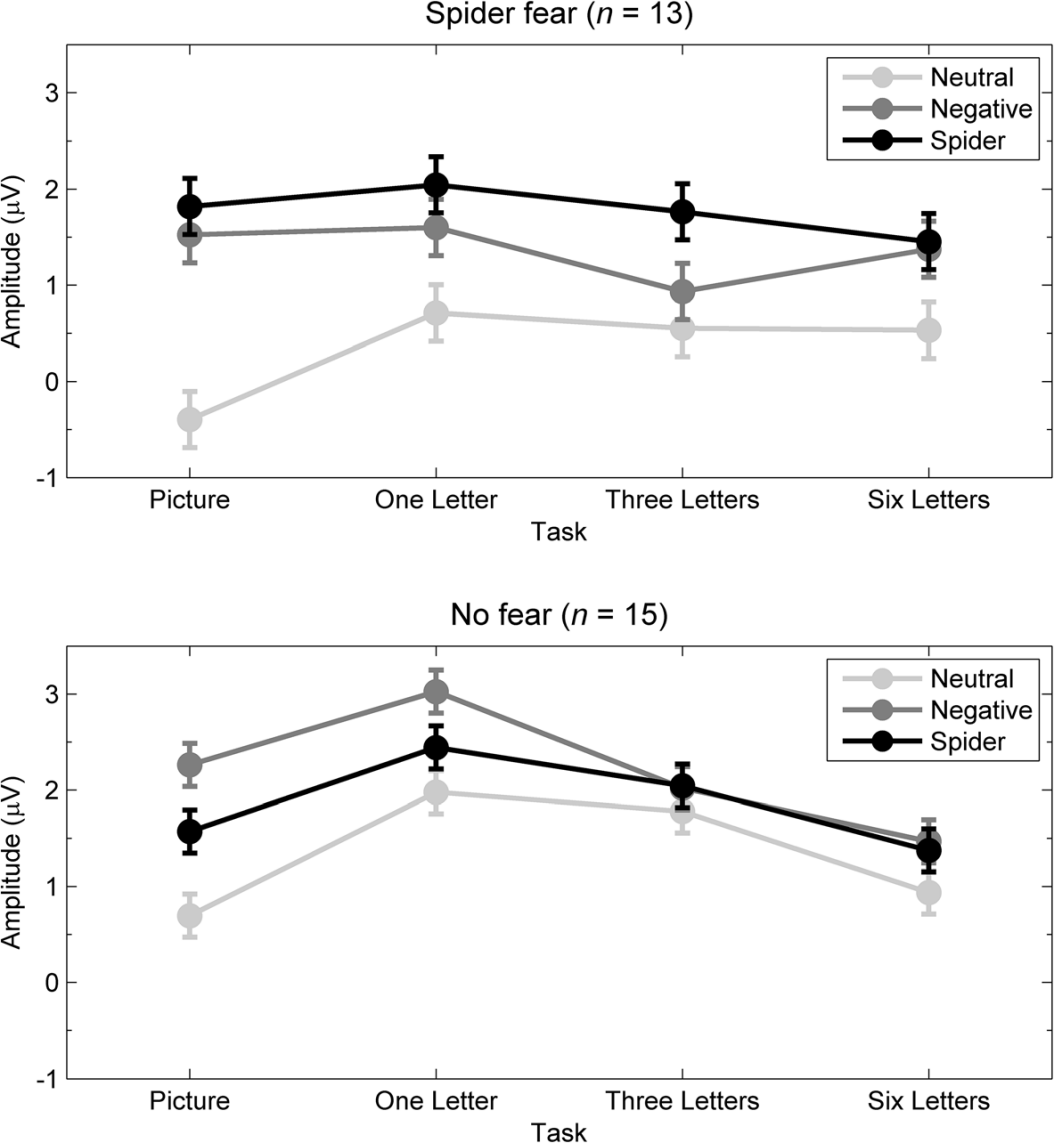
**Mean ERP amplitudes for the LPP.** Mean ERP amplitudes across 300 to 700 ms and across 11 central-parietal electrodes relevant for the late positive potential (LPP) for neutral, negative, and spider pictures during the four tasks, separately for participants with and without spider fear. Error bars refer to the *SEM* derived from the *MSE* within each group.

Figure 3 shows ERP waves for the LPP-relevant electrodes and interval. Figure 4 shows LPP-relevant mean amplitudes to picture type and task separately for fearful and nonfearful participants. Table 1 also shows these mean amplitudes. The ANOVA with group (spider fear, no fear), picture type (neutral, negative, spider), and task (picture, one-letter, three-letter, six-letter) showed mainly an interaction between group and picture type, *F*(2, 52) = 7.00, *p* = .004, *η*^*2*^_   *p*_ = .212. This interaction was caused by more positive amplitudes to spiders (vs. neutral pictures) for fearful than for nonfearful participants, *F*(1, 26) = 21.49, *p* < .001, *η*^*2*^_   *p*_ = .453. This finding indicates that the LPP (i.e., late positive potential) for spiders was larger for fearful than nonfearful participants. In contrast, the LPP for negative (vs. neutral) pictures showed no group differences, *F*(1, 26) < 1, *p* = .60, *η*^*2*^_   *p*_ = .011, and only a general effect across participants, *F*(1, 26) = 36.94, *p* < .001, *η*^*2*^_   *p*_ = .587.

The ANOVA showed no three-way interaction between group, picture type, and task, *F*(6, 156) < 1, *p* = .60, *η*^*2*^_   *p*_ = .027. In contrast, the effect of task yielded a significant two-way interaction between picture type and task, *F*(6, 156) = 5.18, *p* < .001, *η*^*2*^_   *p*_ = .166. The specific contrast with only the picture condition and the six-letter condition (i.e., combined manipulation of attention) showed that the amplitude differences between spiders and neutral pictures decreased from the picture condition to the six-letter condition, *F*(1, 26) = 12.20, *p* = .002, *η*^*2*^_   *p*_ = .319, but this effect did not vary with group, *F*(1, 26) = 2.98, *p* = .096, *η*^*2*^_   *p*_ = .103. Further analyses showed that this task effect (from the picture to the six-letter condition) was caused by manipulations of spatial attention. That is, for the LPP to spiders versus neutral pictures, the contrast of task with only the picture condition and the one-letter condition was significant, *F*(1, 26) = 6.56, *p* = .017, *η*^*2*^_   *p*_ = .201, with no interaction with group, *F*(1, 26) < 1, *p* = .36, *η*^*2*^_   *p*_ = .032. In contrast, the contrast of task that included only the one-letter, three-letter, and six-letter conditions was not significant, *F*(2, 52) < 1, *p* = .63, *η*^*2*^_   *p*_ = .017.

Last, in fearful subjects, mean amplitudes changed similarly for spiders as for negative pictures, *F*(1, 12) < 1, *p* = .57, *η*^*2*^_   *p*_ = .028. That is, the mean LPP to spiders (vs. neutral pictures) was 2.21 during the picture condition and 0.92 during the six-letter condition, and the mean LPP to negative pictures (vs. neutral pictures) was 1.92 during the picture condition and 0.84 during the six-letter condition. Thus, the mean LPP amplitudes decreased from the picture condition to the six-letter condition for spiders (2.21 minus 0.92 = 1.29) and for negative pictures (1.92 minus 0.84 = 1.08), and the mean difference in amplitude change for spiders and negative pictures was 0.21 μV (95% CI = −0.58 to 1.01). The direction of this trend suggested that, if anything, manipulations of attention in fearful subjects *reduced* the LPP more strongly for spiders than for negative pictures.

### Task performance

Over the four tasks (from the picture to the six-letter task), performance decreased gradually (i.e., *d*’ decreased and reaction time increased). In support, the ANOVA of *d*’ with group (spider fear, no fear), picture type (neutral, negative, spider), and task (picture, one-letter, three-letter, six-letter) showed a main effect of task, *F*(3, 72) = 205.10, *p* = .001, *η*^*2*^_   *p*_ = .895. Paired-samples *t* tests between adjacent tasks showed that *d*’ decreased (*p*s < .001) over the four tasks (mean *d*’: picture = 3.94, one-letter = 3.45, three-letter = 2.33, six-letter = 1.55). Similarly, the ANOVA of reaction time with group (spider fear, no fear), picture type (neutral, negative, spider), and task (picture, one-letter, three-letter, six-letter) showed only a main effect of task, *F*(3, 66) = 136.28, *p* = .001, *η*^*2*^_   *p*_ = .861. Paired-samples *t* tests between adjacent tasks showed that reaction time increased (*p*s < .02) over the four tasks (mean reaction times in ms: picture = 544.6, one-letter = 683.2, three-letter = 817.1, six-letter = 863.7). Note that the *df*s vary because response data were missing for two subjects for *d*’ and for four subjects for reaction time due to equipment failure.

### Picture ratings

Table 1 shows mean picture ratings on valence and arousal separately for participants with spider fear and without spider fear. The ANOVA of valence ratings with group (spider fear, no fear) and picture type (spider, negative, neutral) yielded a group by picture type interaction, *F*(2, 52) =21.04, *p* < .001, *η*^*2*^_   *p*_ = .45, and a main effect of picture type, *F*(2, 52) = 49.45, *p* < .001, *η*^*2*^_   *p*_ = .66. Independent-samples *t* tests confirmed that the groups differed only in their ratings of spiders, *t*(26) = 5.02, *p* < .001, *η*^*2*^_   *p*_ = .49. Participants without spider fear rated spider and neutral pictures as equally neutral, *t*(14) < 1, *p* = .50, *η*^*2*^_   *p*_ = .03; and they rated negative pictures as more negative than both spiders, *t*(14) = 6.25, *p* < .001, *η*^*2*^_   *p*_ = .74, and neutral pictures, *t*(14) = 11.94, *p* < .001,*η*^*2*^_   *p*_ = .91. In contrast, participants with spider fear showed similar valence ratings for spiders and negative pictures, *t*(12) = 1.70, *p* = .11, *η*^*2*^_*p*_ = .20, and they rated both spider pictures, *t*(12) = 6.52, *p* < .001, *η*^*2*^_   *p*_ = .78, and negative pictures, *t*(12) = 8.84, *p* < .001, *η*^*2*^_   *p*_ = .87, as more negative than neutral pictures.

The ANOVA of arousal ratings with group (spider fear, no fear) and picture type (spider, negative, neutral) yielded a group by picture type interaction, *F*(2, 52) = 17.28, *p* = .001, *η*^*2*^_   *p*_ = .40, and a main effect of picture type, *F*(2, 52) = 25.77, *p* < .001, *η*^*2*^_   *p*_ = .53. Independent-samples t-tests confirmed that the groups differed only in their ratings of spiders, *t*(26) = 3.55, *p* = .001, *η*^*2*^_   *p*_ = .33. Participants without spider fear rated spider and neutral pictures as equally arousing, *t*(14) < 1, *p* = .57, *η*^*2*^_   *p*_ = .02. They also rated negative pictures as more arousing than both spider, *t*(14) = 5.75, *p* < .001, *η*^*2*^_   *p*_ = .70, and neutral pictures, *t*(14) = 8.98, *p* < .001, *η*^*2*^_   *p*_ = .85. Participants with spider fear showed a trend to rate spiders as more  arousing than negative pictures, *t*(12) = 2.12, *p* = .06, *η*^*2*^_   *p*_ = .27. They also rated spider pictures, *t*(12) = 5.42, *p* < .001, *η*^*2*^_   *p*_ = .71, and negative pictures, *t*(12) = 6.98, *p* < .001, *η*^*2*^_   *p*_ = .80 as more arousing than neutral pictures.

### Questionnaire data

On the spider fear questionnaire (SPQ), participants with spider fear scored higher (*m* = 12.5) than participants without spider fear (*m* = 0.93), *t*(26) = 9.65, *p* = .001, *η*^*2*^_   *p*_ = .78. Also, participants with spider fear felt worse than participants without spider fear after the experiment, as indicated by their higher scores on the PANAS negative subscale (*m* = 13.3 vs. *m* = 10.2), *t*(26) = 2.4, *p* = .022, *η*^*2*^_   *p*_ = .19. No further group differences were observed on the questionnaires.

## Discussion

The main results were that manipulations of attention (spatial attention and perceptual load) decreased the size of the EPN and LPP for spiders (vs. neutral pictures) similarly in spider-fearful and nonfearful participants. Also, for spider-fearful participants, manipulations of attention had similar effects on the EPN and the LPP for spiders as for negative pictures. These results suggest that although spider-fearful participants respond highly emotional to spiders, manipulations of attention reduce motivated attention (as indexed by the EPN and the LPP) similarly for spiders as for other negative pictures.

Although the main results are null findings, they can be informative because they provide interval estimates as well as point estimates of effect sizes [63,64]. For the EPN, a specific ANOVA contrast that tested for a combined effect of attention (i.e., from picture to six-letter tasks) revealed an EPN decrease for spiders (vs. neutral pictures), but this EPN decrease did not vary significantly with group, with a negligible effect size (*η*^*2*^_   *p*_ = .003, *p* = .80). Also, in spider-fearful participants, effects of attention manipulations were similar for spiders as for other negative pictures (*η*^*2*^_   *p*_ = .066, *p* = .38). That is, the mean amplitude change from attention manipulations differed between spiders and negative pictures by only −0.33 μV (95% CI = −1.11 to 0.45). Notably, this trend suggested that in spider-fearful participants, effects of attention manipulations actually decreased the EPN more strongly for spiders than for negative pictures. Also, the upper bound of the confidence interval (0.45 μV) suggests a small maximum effect size for the converse idea that the EPN might actually decrease more strongly for negative pictures than spider pictures. Similarly, for the LPP, the specific ANOVA contrast of the combined effect of attention (i.e., picture vs. six-letter condition) showed an LPP decrease for spiders (vs. neutral pictures), but this LPP decrease did not vary significantly with group (*η*^*2*^_   *p*_ = .103, *p* = .096). Also, in spider-fearful participants, effects of attention manipulations were similar for spiders as for other negative pictures (*η*^*2*^_   *p*_ = .028, *p* = .57). That is, the mean amplitude change from attention manipulations differed between spiders and negative pictures by only 0.21 μV (95% CI = −0.58 to 1.01). As for the EPN, this trend suggested that in spider-fearful participants, effects of attention decreased the LPP more strongly for spiders than for negative pictures. Also, the lower bound of the confidence interval (−0.58 μV) suggests a small maximum effect size for the converse idea that the LPP might actually decrease more strongly for negative pictures than spider pictures. Taken together, these null findings suggest that for spider-fearful participants, manipulations of attention reduce the EPN and the LPP similarly (if not more strongly) for spiders as for other negative pictures.

The present null findings are strenghtened in the context of two manipulation checks. One manipulation check is the finding that across the four tasks, spider-fearful participants clearly differed from nonfearful participants in their emotional responses to spiders and not in their responses to other negative pictures. That is, overall, the amplitude differences between spiders and neutral pictures were larger for fearful participants than for nonfearful participants for the EPN (see Figure 2) and for the LPP (see Figure 4). Similarly, when participants rated the pictures on arousal and valence (at the end of the experiment), spider-fearful participants rated the spiders as more arousing and as more unpleasant than did nonfearful participants. These results replicate previous findings of enhanced EPN and LPP and emotional ratings to spiders in spider-fearful participants pictures [17-27]. Taken together, these findings indicate that our sample of spider-fearful participants responded with strong motivated attention to spiders, that is, they showed large emotional responses in terms of ERP measures and in terms of self-reported valence and arousal ratings.

Another manipulation check is the finding that across participants, manipulations of attention reduced the EPN and the LPP to spiders (vs. neutral pictures). Previous studies reported that the LPP to spiders (vs. neutral pictures) was enhanced not only in fearful participants but also in nonfearful participants [17-19,21]. The present results extend these findings because they indicate that manipulations of attention reduce the EPN and LPP to spiders across participants. As shown in Figure 2, the combined effects of spatial attention and perceptual load reduced the EPN amplitude differences between spiders and neutral pictures. That is, from the picture task to the six-letter task, the negative amplitude difference (i.e., early posterior negativity) between spiders and neutral pictures decreased across participants (with no group difference, as discussed above). This effect of task was apparently caused by a combination of spatial attention and perceptual load, as follow-up analyses that tested specifically for effects of spatial attention (i.e., picture vs. one-letter task) and for effects of perceptual load (i.e., one-letter, three-letter, and six-letter tasks) were not significant. As shown in Figure 4, the combined effect of spatial attention and perceptual load also reduced the LPP amplitude difference between spiders and neutral pictures. That is, from the picture task to the six-letter task, the positive amplitude difference (i.e., late positive potential) between spiders and neutral pictures decreased across participants (with no group difference, as discussed above). However, this effect of task was caused by spatial attention, as follow-up analyses that tested specifically for effects of spatial attention (i.e., picture vs. one-letter task) and for effects of perceptual load (i.e., one-letter, three-letter, and six-letter tasks) were significant only for effects of spatial attention. In sum, the present results that the EPN and LPP for spiders were reduced by manipulations of attention replicate and extend evidence of reduced EPN and LPP to emotional (vs. neutral) pictures when voluntary attention is directed away from the pictures [30,34-43].

## Conclusions

The present results suggest that although spider-fearful participants respond highly emotional to spiders, manipulations of attention reduce motivated attention (as indexed by the EPN and the LPP) similarly for spiders as for other negative pictures. These results indicate that motivated attention to spiders in spider fear is not protected again manipulations of attention. Thus, the findings extend previous reports that showed similar effects with generally emotional pictures (from the IAPS set). Further, these findings imply that attention to spiders in spider fear does not exceed the level of attention expected from the spider pictures’ high arousal and negative valence (i.e., their intrinsic motivated attention).

## Competing interests

The authors are unaware of any competing interests.

## Authors’ contributions

JN and SW designed the study, analyzed the data, and wrote the paper. JN collected the data. Both authors read and approved the final manuscript.
